# Effect of acute stressor on reproductive behavior differs between urban and rural birds

**DOI:** 10.1002/ece3.2347

**Published:** 2016-08-20

**Authors:** Mikus Abolins‐Abols, Sydney F. Hope, Ellen D. Ketterson

**Affiliations:** ^1^ Department of BiologyIndiana UniversityBloomingtonIndiana; ^2^ Department of Fish and Wildlife ConservationVirginia TechBlacksburgVirginia

**Keywords:** Aggression, behavioral plasticity, flexibility, global change, reproduction, stress

## Abstract

The life‐history trade‐off between self‐maintenance and reproduction posits that investment in one function decreases investment in the other. Manipulating the costs and benefits of functions involved in a trade‐off may alter this interaction. Here we ask whether investment in self‐maintenance during a stress response alters territorial behavior in wild Dark‐eyed Juncos and whether rural and urban birds, which are known to differ in the magnitude of the stress response (greater in rural), also differ in the degree to which stress reduces territorial behavior. In rural and urban habitats, we measured territorial behavior using song playback, followed by either an acute stressor (capture and collection of a blood sample) or a nonstressful control situation. The following day, we again measured territorial behavior, predicting greater reduction in territorial behavior in individuals exposed to the stressor but a lesser reduction in territorial behavior in the urban as compared to the rural environment. We further assessed individual and population differences in response to stressors by measuring flight initiation distance, breath rate, and corticosterone levels in the blood. The rural population had a higher physiological and behavioral stress response than the urban population, and acute capture stress had a lasting (24 h) negative effect on territorial behavior, but only in the rural habitat. However, individual‐level differences in measures of the stress response did not explain variation in the impact of stress on territorial behavior. Our findings show that stressors can have a negative effect on territorial behavior, but that this effect may differ between populations that vary in their stress ecology.

## Introduction

Perhaps the most fundamental life‐history trade‐off is that between self‐maintenance and reproduction because it integrates two major aspects of fitness: survival and reproductive success (Williams 1966; Stearns 1989, 1992; Reznick et al. 2000; Zera and Harshman 2001; Wingfield and Sapolsky 2003; Deviche et al. 2012). This trade‐off is critical in explaining the diversity in life‐history patterns of animals across taxa and environments (Rose 1983; Stearns 1989, 1992). In each environment, individuals must reach a particular balance in allocation of time and energy to reproduction and self‐maintenance in order to maximize their lifetime reproductive success (Rose 1983; Stearns 1989, 1992). An indispensable component of life‐history trade‐offs is behavior, because many decisions about survival and reproduction happen in real time (Houston and McNamara 1999). By changing their behavior, animals can quickly adjust investment of time and energetic resources to competing life‐history functions. Understanding how behavior mediates investment in reproduction and self‐maintenance in changing environments is important, especially in the context of current habitat and climate change.

An important component of the reproductive phenotype is territorial behavior. While territorial behaviors differ between taxa, territory defense often represents investment in current reproduction at the expense of survival and self‐maintenance: territorial behaviors enable or secure access to mates and resources but are costly, risky, and time‐consuming (Davies and Halliday 1978; Clutton‐Brock and Albon 1979; Stamps 1994; Houston and McNamara 1999; Moller et al. 2007). This is especially evident in songbirds, where males advertise and defend their territory throughout the day across the breeding season using songs, visual displays, and physical attacks to ward off intruders (McGregor Peter 1993; Searcy et al. 2006; Derryberry 2007; Searcy and Beecher 2009; Atwell et al. 2014).

The trade‐off between reproduction and self‐maintenance implies that an increase in investment in one of these functions will lead to a decrease in the other. If an environment is risky or stressful, territorial behavior is predicted to decrease compared to a more benign environment, increasing the probability of survival at the cost of current reproduction. Studies of captive animals have supported this prediction, showing that predation risk, food, and restraint stress down‐regulate reproductive behaviors, including aggression toward other males, courtship, and copulatory behaviors (Menendez‐Patterson et al. 1980; Bell and Sih 2007; Lynn et al. 2010). In the wild, natural stressors, such as simulated predation risk, have been shown to have a negative effect on showy territorial behavior (Akçay et al. 2016) and reduce the number of offspring produced per year (Zanette et al. 2011).

A potential mediator of reduction in reproductive behaviors is the physiological stress response, which plays a crucial role in self‐maintenance. The stress response involves two main hormonal axes, the adrenergic system and hypothalamic–pituitary–adrenal (HPA) axis, which control release of corticosterone (CORT), a major vertebrate stress hormone (Romero and Butler 2007). These axes regulate metabolism and flight response, enabling appropriate responses to short‐ and long‐term challenges to survival or homeostasis (Romero and Wingfield 2015). Both the adrenergic system and the HPA axis have been shown to negatively affect reproductive function (Wingfield and Sapolsky 2003; Chand and Lovejoy 2011). For example, CORT can negatively affect function of gonads and reproductive centers in the brain (Viau 2002; Wingfield and Sapolsky 2003; Dong et al. 2004; Gore et al. 2006), serving as a physiological brake on reproductive physiology and behavior.

Studies showing that reproductive behaviors or physiology decrease in response to stressors are informative (Pickering et al. 1987; Moore et al. 1991; Deviche et al. 2010; McGuire et al. 2013; Lynn et al. 2015), but often miss the complexity of the interaction between the stress response and reproduction as seen in the wild (Chand and Lovejoy 2011). In the wild, the costs and benefits of territorial behavior and self‐maintenance differ between habitats or across time, which is likely to select for differences in how investment in one function affects the other.

One of the main environmental processes that may affect the balance between reproduction and self‐maintenance is human‐induced rapid environmental change (HIREC; Sih et al. 2011). Habitat change due to urbanization is an especially potent selective force, as urban and rural areas differ in resource availability, microclimate, habitat structure, and disturbance levels (Gilbert 1989). Any one of these differences has the potential to substantially alter life histories of animals.

For example, greater levels of disturbance in urban habitats might chronically activate physiological stress response, resulting in detrimental effects on animal fitness if no acclimation occurred (Romero and Wingfield 2015). Some urban populations show a reduced corticosterone (CORT) increase in response to a stressor, compared to rural animals, which may act to reduce long‐term costs of the stress response in urban environments (Partecke et al. 2006; Atwell et al. 2012; but see Fokidis et al. 2009; Bonier 2012). Whether or not changes in the stress response also result in reduced effects of stressors on reproductive phenotype is poorly understood (Bonier 2012). Rural and urban populations also show differences in reproductive behaviors (Newman et al. 2006; Mockford and Marshall 2009; Evans et al. 2010; Fokidis et al. 2011; Atwell et al. 2012) and life history (Partecke and Gwinner 2007; Atwell et al. 2014), suggesting that urban–rural comparisons can serve as a strong model in which to study how the interaction between stress and reproduction changes across environments.

**Figure 1:**
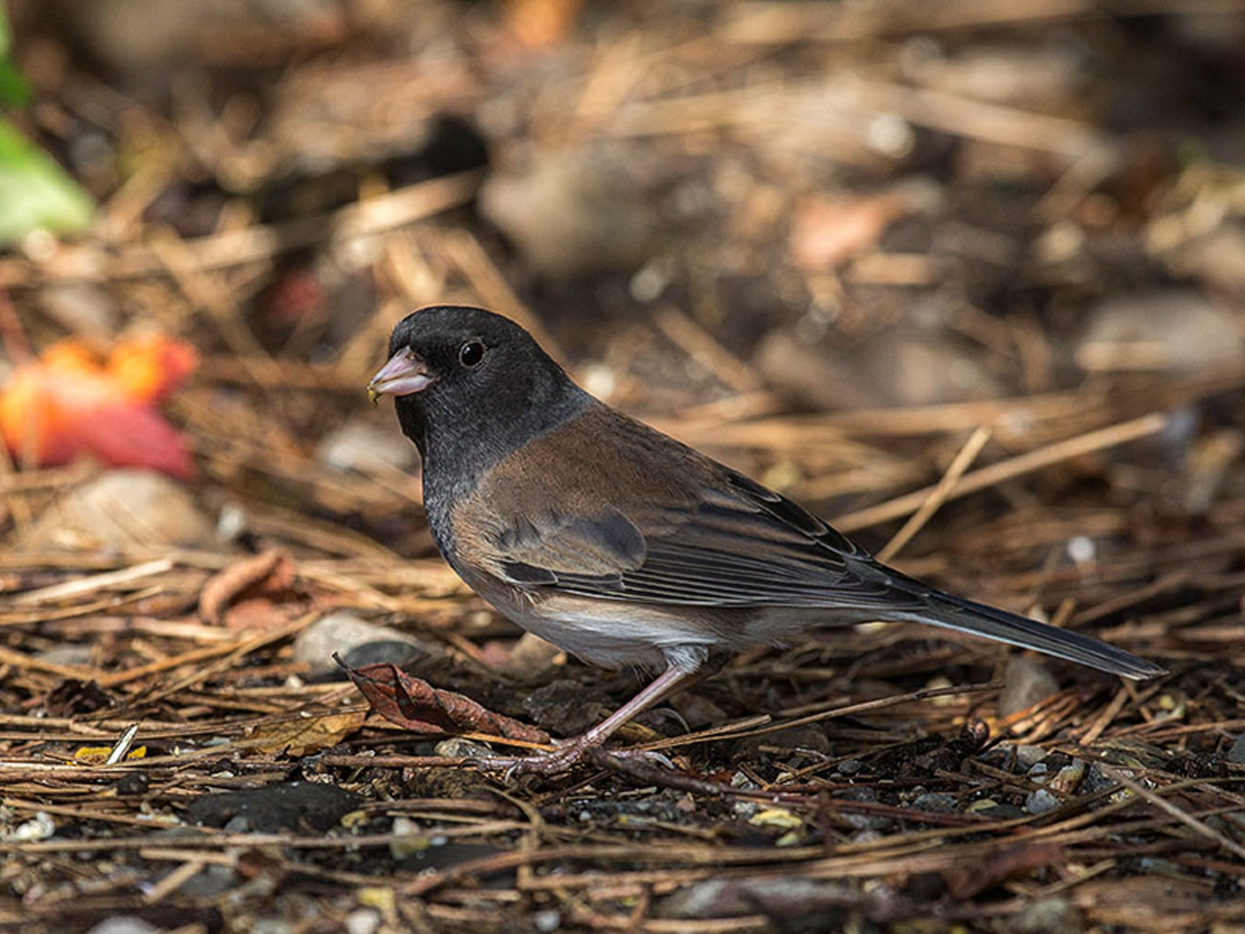
Male Oregon Junco (*Junco hyemalis*).Photograph by Joseph Higbee.

In this study, we asked first whether acute stress has a negative effect on territorial behavior in wild male Oregon Juncos (*Junco hyemalis,* Linnaeus, Fig. 1), second, whether any effect would vary between urban and rural populations, and third, whether differences in the stress response between individuals and habitats could explain variation in the effect of stressors on territorial behavior. We predicted that acute stress would decrease territorial displays, but that the magnitude of the impact on territorial behavior would depend on the magnitude of the stress response. In particular, we predicted that urban individuals, which have been shown to have a lower physiological stress response, would show a reduced effect of a stressor on territorial behaviors compared to a rural population with a higher stress response.

## Methods

### Organism and location

We studied urban and rural Oregon Juncos, a common songbird in southern California, during the breeding season from 13 May through 14 June 2013. Urban birds (n=69) were studied on the University of California Los Angeles (UCLA) campus in Los Angeles, a site that was colonized by juncos within the last two decades (J. Diamond and K. Garrett pers. comm.). Rural birds were studied around the Buckhorn Campground (*n* = 50) and Charlton Flats Picnic area (*n* = 34) in the Angeles National Forest. These sites were 10.3 km apart, in the conifer forest (elevation 1500–2000 m), and were the closest accessible natural junco habitat to Los Angeles (46.7 km from UCLA). Neither rural nor urban population had been studied before. We studied territorial behavior and stress response in overlapping sets of individuals: some but not all birds from the territorial study were also part of a larger study on urban–rural differences in stress response. All individuals, except control individuals for the behavioral study, were individually marked with unique set of color bands and USFW metal bands.

### Territorial behavior: trials

Territories of free‐living junco males (39 urban and 45 rural birds) were mapped by observing natural singing perches and using song playback. We quantified male territorial behavior using 5‐min‐long simulated territorial intrusions (STI) between 7:00 and 13:00 in the estimated center of male's territory over 25 days between May 13 and June 14. One to nine STIs (median 5) were conducted per day. Territories where STIs were conducted on a given day were at least one territory apart to minimize the effect of a nearby STI on focal male's behavior. For each male, we conducted a baseline STI before the treatment and a second post‐treatment STI the next day to measure change in behavior due to treatment. During an STI, the focal male was presented with a live male junco in a small cage. Simultaneously, a speaker next to the cage was broadcasting randomly selected Oregon junco male long‐range songs, following previously established protocols (McGlothlin et al. 2007). Lure birds (*n* = 4) and songs (*n* = 12) were randomized between trials to avoid pseudoreplication. In post‐treatment trials, males were never presented with the same STI tape to avoid habituation. During each 5‐min trial, we recorded number of flights, estimated the distance of the focal male from the lure (estimates were later checked using a tape measure), and measured duration of singing every 15 sec. We then calculated median distance from the intruder, time to closest approach, and the amount of time males spent singing. Distance from the intruder and song have been previously shown to serve as aggressive signals in passerines (Searcy et al. 2006; Derryberry 2007), and flight behavior is often used as a measure of exploration (Dingemanse et al. 2004).

### Territorial behavior: stress treatments

Males were randomly assigned to control or acute stressor treatments (*n* = 41). In both treatments, we measured male territorial behavior on two consecutive days. In the control treatment, males were not disturbed in‐between the measurements. This treatment accounted for possible change in behavior due to habituation (Evans et al. 2010) or winner/loser effects in repeated territorial behavior measurements (Chase et al. 1994). These birds were not handled at any point during the season and were unbanded. We ensured that the same male was present in the consecutive trials, by noting individual song and plumage characteristics and excluding all trials that attracted more than one male or where we otherwise were not confident that the two trials were conducted on the same males.

In the stressor treatment, males were captured following the first behavioral trial using mist nets and retained for an hour in paper bags, a standard acute stressor treatment (Wingfield and Ramenofsky 1999) and then were released. During the restraint, birds were banded and blood was taken for hormone analyses (see below).

### Stress response: blood sampling

As part of a larger hormonal study in the same populations (M. Abolins‐Abols and E. D. Ketterson, unpubl. ms.), we collected blood from birds in the acute stressor treatment (*n* = 28) as well as other birds (*n* = 46) that were not part of the behavioral study. We collected peripheral blood samples at 3 and 50 min after capture from the brachial vein to measure baseline corticosterone (CORT) and testosterone (T) as well as stressor‐induced changes in CORT (50 − 3 min levels). Blood was collected using heparinized microcapillary tubes and stored on ice until they were centrifuged 2–6 h later to separate blood cells and plasma, which was then stored at −20°C. After the last blood sample, we used calipers to measure the volume of the cloacal protuberance, a sperm storage organ.

### Stress response: hormone assays

We used diethyl ether to extract CORT and T from plasma, measuring the efficiency of extraction (mean 96.0%) with tritiated corticosterone. CORT levels were then analyzed with EIA (Cayman Chemical Company,Cat. no. 500655 Ann Arbor, MI, USA). Samples were randomly distributed across 10 plates (within‐plate CV: 8.56%; between‐plate CV: 7.35%). Baseline plasma T concentration was analyzed using EIA (Enzo Life Sciences,Cat. no. ADI‐901‐65 Farmingdale, NY, USA). Samples were randomly distributed across 12 plates (within‐plate CV: 8.076%; between‐plate CV: 17.79%). Hormone levels were determined in reference to eight‐point (CORT) or nine‐point (T) standard curve using a cure‐fitting program (Microplate Manager, Bio‐Rad Laboratories Inc., Hercules, CA). Samples were corrected for extraction efficiency. Both assays have been previously validated in our laboratory.

### Stress response: behavioral measures

Breath rate has been associated with the heart rate and is used to noninvasively characterize differences in parasympathetic nervous system reactivity to stressors (Koolhaas et al. 1999; Carere and van Oers 2004; Krams et al. 2014). We measured breath rate in the captured birds from the acute stressor treatment of the territorial behavior study (*n* = 27) as well as other birds in the populations (*n* = 46) by counting the number of chest movements during a 10‐sec period after one hour of restraint. We assessed flight initiation distance (FID), a common measure of fearfulness (Stankowich and Blumstein 2005) of adult (*n* = 45) and juvenile (*n* = 46) birds from both urban and rural populations by measuring the distance with a tape measure at which a bird first fled from the observer (SFH) approaching it at a normal walking pace (Atwell et al. 2012). We also measured FIDs of birds in the acute stressor treatment shortly before capture (*n* = 25). We used these observations to investigate the relationship between territorial behavior and the stress response.

### Statistical analysis

#### Territorial behavior

All statistical analyses were conducted in R 3.0.2 (R Core Team 2013). Because we measured multiple aspects of territorial behavior (approach distance, singing, and flights), we used multivariate tests to investigate the effects of population and stress treatment on behavior as a whole. Differences in baseline territorial behavior (behavior on day 1) were analyzed using nonparametric multivariate tests (PerMANOVA, package *vegan*; Oksanen et al. 2015), followed by analysis of each behavior separately using univariate tests (Wilcoxon Signed‐Rank (WSR) or *t*‐test). The effect of the stressor on behavior was calculated by subtracting the baseline territorial behavior from the post‐treatment behavior. We chose this approach instead of using the baseline behavior as a covariate because using change as the dependent variable allowed us to directly investigate the effect of stress and population on behavioral change. Behavioral change measures were approximately normally distributed for all three individual behaviors, allowing us to use parametric tests, which, given the low sample size, increased our power to detect significant effects. We used two‐way multivariate parametric tests (MANOVA, package *car*; Fox and Weisberg 2011) with type III sums of squares to assess the effect of population, treatment, and their interaction on behavioral change. The residuals satisfied parametric requirements. We then conducted one‐way MANOVAs to investigate the factors driving the interaction.

#### Stress response (population comparison)

Stress‐induced CORT levels were calculated by subtracting the 3‐min baseline from stress‐induced 50 min value. We used mixed‐effects models (LMM, package *nlme*; Pinheiro et al. 2015) with individual as a random factor to analyze population differences in hormone levels, breath rate, and FID. Models of stress response included date as a covariate.

#### Stress response and territorial behavior

We used MANOVA (package *car*) with type III sums of squares to investigate the relationship between individual differences in stress response measures and territorial behavior in birds from the acute stressor treatment. Models included population as a covariate.

## Results

Urban birds differed from rural birds in their response to STI (PerMANOVA, *n* = 78, *F* = 6.749, *P* = 0.003). Analysis of each behavior separately revealed that urban birds approached the lure significantly more closely (WSR, *n* = 84, *W* = 1229.5, *P* < 0.001) and sang significantly less (WSR, *n* = 84, *W* = 1193.0, *P* = 0.002) than did rural birds. They also approached the STI stimulus sooner (WSR, *n* = 84, *W* = 1119.5, *P* = 0.028). Urban and rural birds did not differ in the number of flights (*t*‐test, *n* = 84, *t* = −0.078, df = 64.399, *P* = 0.938).

**Figure 2:**
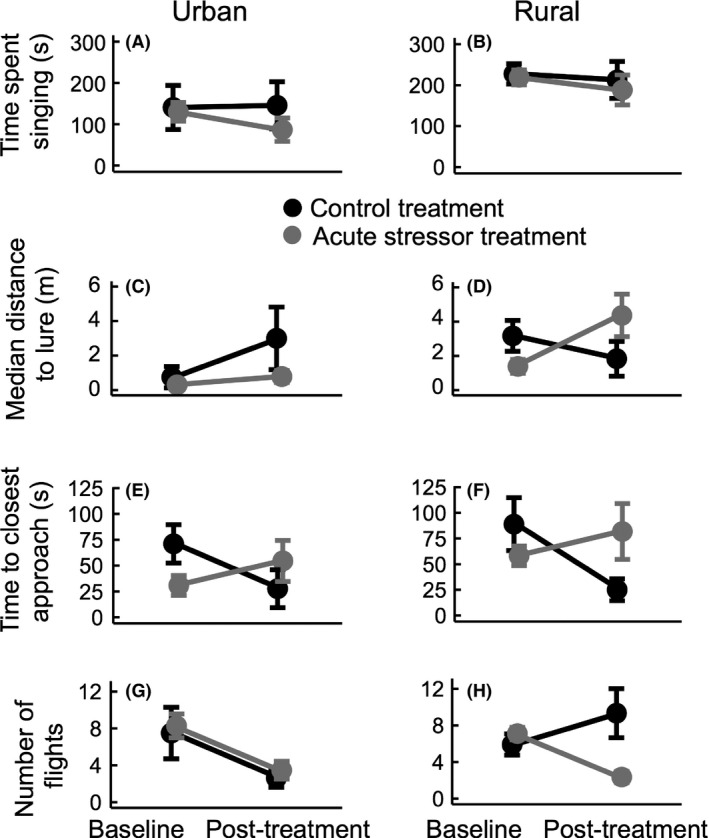
Exposure to an acute stressor affects territorial behavior differently in rural and urban birds. Circles and error bars denote means and standard errors of territorial behaviors during the pretreatment and the post‐treatment STI in control (black) and acute stressor (gray) treatments. An acute stressor did not affect singing in either population (A, B), although rural birds sang more in general. Treatment did not have a significant effect on median approach distance in urban birds (C), whereas in rural bids, stress treatment led to an increase in median approach distance while control birds did not change their behavior (D). Stress treatment had a similar effect on approach time in both urban (E) and rural (F) populations – in both cases, birds exposed to the stressor approached the lure more slowly, whereas control birds approached the lure more rapidly. The stressor had a different effect on number of flights in urban (G) and rural (H) birds: in the urban population, birds from both treatments reduced number of flights, whereas in the rural population, control birds increased while birds exposed to the stressor decreased number of flights.

We found that the stressor treatment had a significant effect on territorial behavior as a whole, but that this effect differed between populations (MANOVA, *n* = 41, site‐by‐treatment interaction: *F*_4,28_ = 3.305, *P* = 0.024, Fig. 2). To unravel this interaction, we conducted one‐way MANOVA tests to evaluate the effect of treatment within single populations. We found that exposure to an acute stressor led to a significant reduction in territorial behavior in the rural population between day 1 and day 2 as compared to controls (MANOVA, *n* = 19, *F*_4,10_ = 4.599, *P* = 0.023). However, the acute stressor did not affect territorial behavior in the urban birds (MANOVA, *n* = 22, *F*_4,15_ = 1.356, *P* = 0.296).

**Figure 3:**
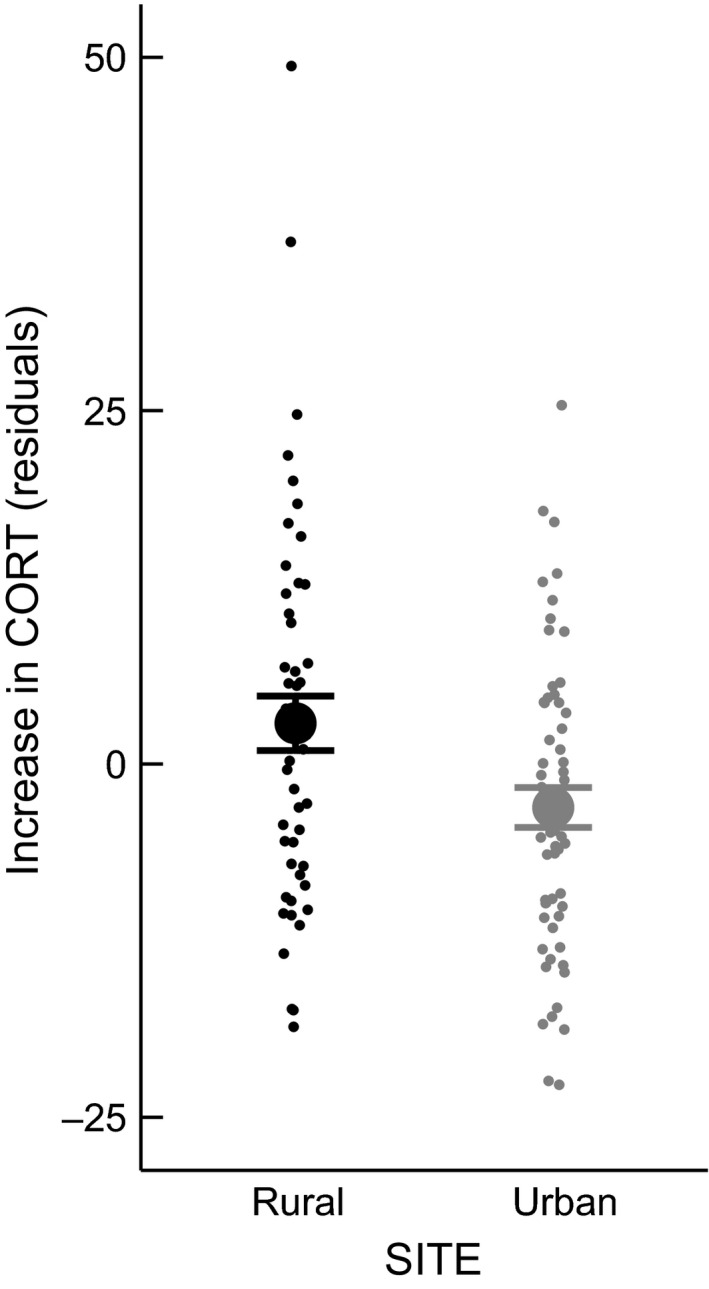
Stressor‐induced CORT is higher in the rural population than in the urban population. CORT values are corrected for the effect of date (both populations show decrease in stressor‐induced CORT (ng/ml) across the breeding season).

**Figure 4:**
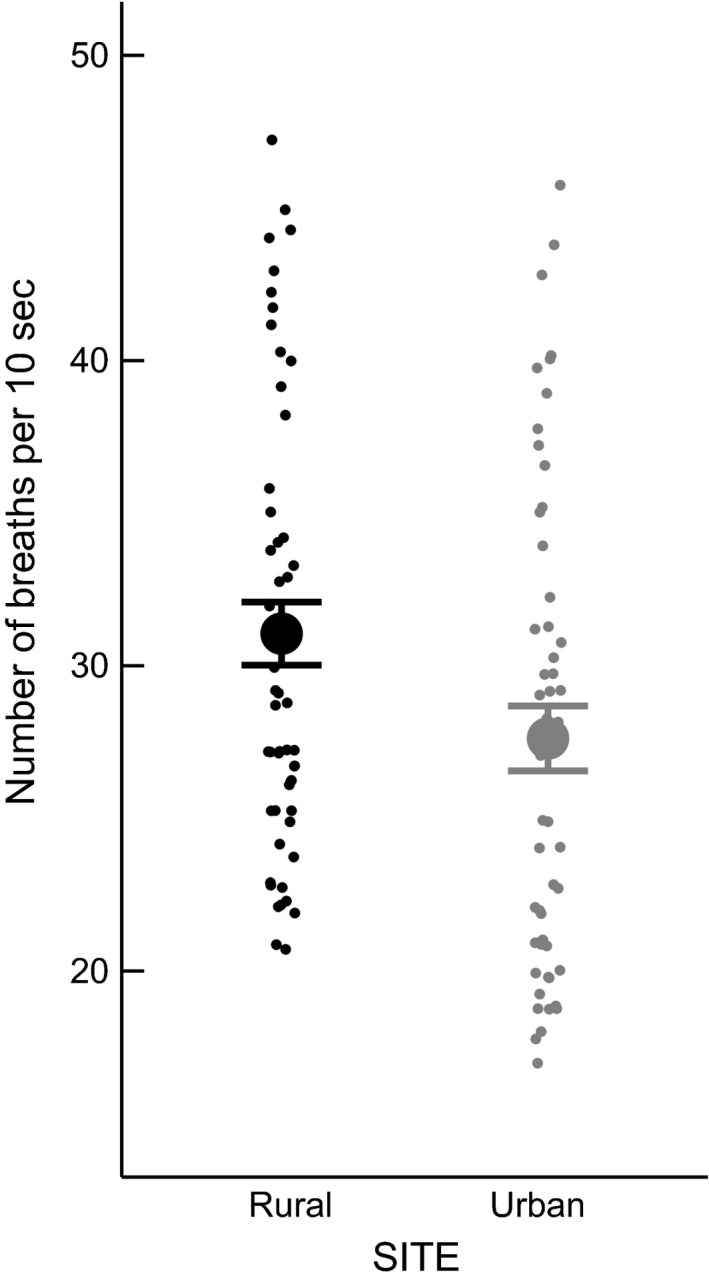
Breath rate after handling was higher in the rural than urban population. Graph shows means ± SEs. Points are jittered to aid interpretation.

**Figure 5:**
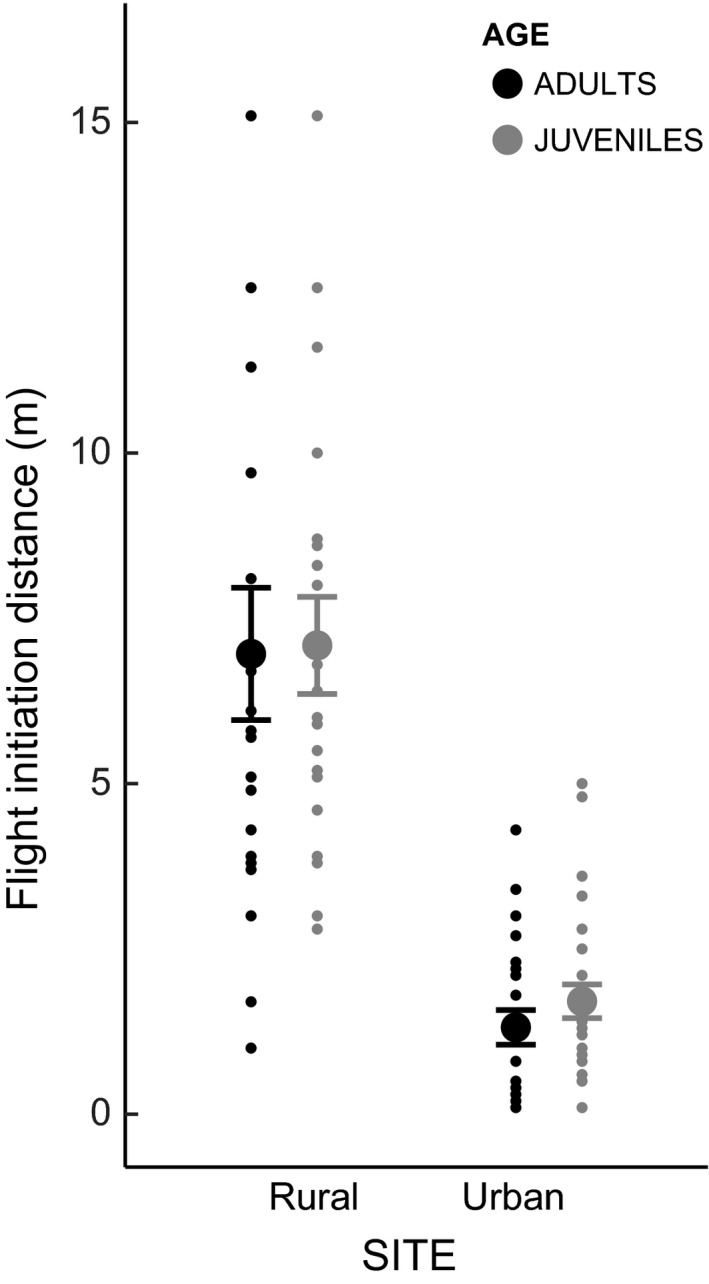
FIDs were lower in the urban population compared to rural population in both adult (black) as well as juvenile (gray) birds. Graph shows individual data points and means ± SEs.

Urban and rural populations did not differ in baseline CORT levels (LMM, *n* = 107 *F*_1,16_ = 1.123, *P* = 0.305), but urban birds showed significantly lower stress‐elevated CORT levels than rural birds (LMM, *n* = 107, *F*_1,16_ = 5.957, *P* = 0.027, Fig. 3). Urban individuals had lower breath rate (LMM, *n* = 105, *F*_1,88_ = 4.614, *P* = 0.0345, Fig. 4) and showed shorter FIDs (ANOVA, *n* = 91, *F*_1,87_ = 80.2097, *P* > 0.001) than rural birds, a difference that was also present in juvenile birds (Fig. 5). None of the measures of stress response were correlated with each other.

Urban and rural birds did not differ in baseline T levels (LMM, *n* = 86, *F*_1,71_ = 2.632, *P* = 0.109) or in the volume of their cloacal protuberance (LMM, *n* = 134, *F*_1,24_ = 0.572 *P* = 0.572).

Neither baseline (MANOVA, *n* = 28, *F*_4,17_ = 0.358, *P* = 0.835) nor stress‐induced corticosterone levels (MANOVA, *n* = 28, *F*_4,17_ = 0.824, *P* = 0.528) explained variation in reduction of territorial behavior in individuals. Differences in change in territorial behavior were not related to breath rate (MANOVA, *n* = 26, *F*_4,17_ = 0.40188, *P* = 0.805) or FID (MANOVA, *n* = 37, *F*_4,28_ = 0.82552, *P* = 0.520).

## Discussion

In this study, we investigated whether territorial behavior decreases in response to an acute stressor and whether the effect of a stressor on territorial behavior is different between individuals and populations that differ in their stress response. We found that an acute stressor has a long‐term (≥24 h) effect on territorial behavior, but that this effect differed between rural and urban birds. Rural birds reduced their territorial behavior in response to the stressor, whereas urban birds did not. The populations also differed in their stress response: urban birds showed lower stress‐induced CORT, shorter FIDs, and had a lower breath rates. However, we did not find an association between individual variation in stress response and behavior: neither baseline nor stress‐induced corticosterone, nor breath rate, or FID significantly predicted individual‐level change in territorial behavior in response to a stressor.

Our findings demonstrate that stress can have a significant negative effect on territorial behavior in the wild. These findings are on a par with other studies that have shown a negative effect of stressors on reproductive phenotype (Menendez‐Patterson et al. 1980; Bell and Sih 2007; Deviche et al. 2010; Lynn et al. 2010) and suggest that an increase in environmental stressors may alter (decrease) reproductive behaviors in wild populations. Territorial behavior is essential for reproductive success (Stamps 1994) and affects survivorship (Marler et al. 1995). Therefore, a stress‐induced decrease in behavior may be important for population dynamics and viability (Fefferman and Romero 2013; Wingfield 2013) and have evolutionary consequences. Importantly, this effect may not necessarily be negative – a decrease in territorial behavior may increase the survivorship of animals in dangerous environments. The effect of the change in territorial behavior on reproductive success may be more complex, as reproductive success depends on both an individual's territorial behavior and the territorial behavior of others (Dugatkin and Reeve 1998). If all individuals respond to stressors the same way, relative reproductive success of each individual may remain the same across different stress environments. However, individuals often vary consistently in their stress response (Romero and Reed 2008; Rensel and Schoech 2011) and stressors often increase phenotypic variance in a population (Badyaev 2005). A stress‐induced increase in the variance of reproductive success, such as when only high‐quality individuals can afford to be territorial and reproduce, may lead to low effective population size, reducing the capacity to respond to evolutionary change (Hedrick 2005).

Our results comparing populations, however, suggest that the sensitivity of reproduction to stress can vary between populations in close proximity. It is not clear whether the urban–rural difference in the sensitivity of territorial behavior to stress is an adaptive response to population differences in environmental stressors or other aspects of environment. Regardless, our results suggest that urban birds may be more vulnerable to predation than rural birds, as they did not reduce territorial behavior following a major threat to their homeostasis and allowed closer approach of humans before fleeing. In terms of reproductive success, however, an urban phenotype may allow birds to retain territory and mate in the face of frequent disturbance. In the life‐history framework, these differences suggest that urban populations generally may invest less in self‐maintenance and more in reproductive function than rural birds (but see Sol et al. 2012 for species differences). While we do not know whether our findings can be generalized across other urban and rural populations and species, juncos across California show parallel divergence in multiple other aspects of phenotype in comparison with their rural counterparts: urban juncos consistently have a lower stress response, duller plumage coloration, and an advanced breeding season (Yeh and Price 2004; Atwell et al. 2014). Furthermore, urban–rural differences in baseline territorial behavior shown in our study are consistent with differences between other rural and urban junco populations (Newman et al. 2006; Atwell et al. 2014). Similarities exist across species too: many urban birds populations show advanced reproductive phenology (Deviche and Davies 2013), different territorial behavior (Fokidis et al. 2011), and an altered stress response (Partecke et al. 2006; Atwell et al. 2012; but see Fokidis et al. 2009; Bonier 2012). Whether these similarities suggest a general urban physiological and behavioral “syndrome” is not clear and warrants further research.

The mechanisms that mediate the differences in reduction of territorial behavior in response to an acute stressor, however, remain unclear. Reduction in territorial behavior in response to capture stress in the rural population might be due to a strong or long‐lasting stress‐induced physiological “brake” on reproduction, in contrast to a relaxed or short‐lived cross‐talk between the stress response and reproductive function in urban birds. Alternatively, rural birds might be better at learning to associate an aggressive encounter (STI) with danger of capture than urban birds. We could only test for differences in the strength of stress response (the “brake”) between populations and individuals. Urban birds showed lower stress‐induced increase in CORT as well as breath rates and FIDs than rural birds. However, we did not find any association between individual differences in the magnitude of the hormonal and behavioral stress response and decrease in territorial behavior, although this does not preclude the possibility that stress response may explain variation in behavior at other time points.

The lack of association between stress response measures and territorial behavior could be due to the fact that our measures of stress response are measuring other aspects of physiology and behavior. For example, CORT physiology is often linked to metabolism and food availability (Moore et al. 2000; Duckworth et al. 2001); therefore, it might be reflecting differences in resource availability between urban and rural areas, not differences in stressors. Even if CORT measures reflect differences in stress response, peripheral hormones often explain little if any variation in behavior (Fokidis et al. 2011; but see Bergeon Burns et al. 2013; Atwell et al. 2014), as the signaling of those same hormones in the brain can be decoupled from the periphery (Schlinger et al. 2001). It is also not completely clear what aspects of physiology or stress response are reflected by breath rate and FID measures (Réale et al. 2010). FID and breath rate have often been found to differ between animals of different behavioral “personality” phenotypes (Carere and van Oers 2004; Stankowich and Blumstein 2005; Krams et al. 2014), but it is not clear whether they represent context‐specific or generalized responses to stimuli (e.g., FID may reflect acclimation to humans, but not other stressors). Population and individual variation in reduction of territorial behavior in response to an acute stressor in juncos may thus be due to either aspects of stress response that we did not measure such as the magnitude of the adrenergic response, differences in the recovery and duration of a stress response (Wingfield 2013), or differences in learning or habituation. Distinguishing among these hypotheses would allow us to better understand how animals adapt to novel environments.

In conclusion, we have shown that an acute stressor leads to a decrease in territorial behavior of dark‐eyed juncos in rural areas. Reduction of territorial behavior due to environmental stressors may have significant consequences for population ecology. However, the urban–rural difference suggests that the effect of stress on reproduction is dynamic. Parallel differences in other aspects of stress response between these and other urban and rural junco populations suggest that the decreased sensitivity of reproductive phenotype to stressors in urban birds in Los Angeles may represent a generalized change in life‐history strategies of urban animals, whereby urban birds invest less in self‐maintenance and more in reproduction. To test this hypothesis, more studies are needed that investigate the interaction between reproduction and stress response across different stress environments. Uncovering the mechanisms that mediate this interaction will further aid in our understanding of how animals adapt to increasingly anthropogenic environments.

## Conflict of Interest

The authors do not have any conflict of interest to declare.

## Data Accessibility

Data analyzed in this article are available at the Dryad Digital Repository as doi: http://dx.doi.org/10.5061/dryad.46r48.
